# Modifying Antifungal Peptides as Safe Food Preservatives

**DOI:** 10.1021/acs.jafc.5c02065

**Published:** 2025-07-10

**Authors:** Eyal Simons, Idan Yakir, Einav Malach, Aygun Israyilova, Zvi Hayouka

**Affiliations:** † Institute of Biochemistry, Food Science and Nutrition, The Robert H. Smith Faculty of Agricultural, Food & Environment, 62384 The Hebrew University of Jerusalem, Rehovot 76100, Israel; ‡ Singapore-HUJI Alliance for Research and Enterprise (SHARE), The Cellular Agriculture (CellAg) Programme, Campus for Research Excellence and Technological Enterprise (CREATE), Singapore 138602, Singapore; § Laboratory of Microbiology, Center for Excellence in Research, Development & Innovation, Baku State University, Z. Khalilov 33, Baku AZ1148, Azerbaijan

**Keywords:** antimicrobial peptide, antifungal peptide, food preservation, food safety, fungal contamination, *Penicillium expansum*

## Abstract

Food loss and waste (FLW) constitute a major global concern with significant environmental and economic ramifications. A major cause of FLW is fungal contamination, leading to food spoilage. Here, we investigate the use of antimicrobial peptides (AMPs) as a new source for food preservatives to combat filamentous fungi and yeast contamination. Specifically, we focused on two model AMPs, BP100 and P-113, and their derivatives that were designed and synthesized as antifungal agents for food preservation. BP100 and P-113 were modified in order to enhance their efficacy. BP100 was modified by substituting its N-terminal lysine with tryptophan (i.e., BP100 K1W) to extend its hydrophobic α-helical side, and P-113 was extended by synthesizing the original sequence triplicated (i.e., P-113 Triple). The modified peptides demonstrated strong antifungal and antibacterial activities. Importantly, they showed low cytotoxicity and were digestible, indicating their safety for consumption. BP100 K1W was particularly effective in a hummus food matrix model, successfully inhibiting ’s growth and consequently extending the food’s shelf life. Thus, this highlights its potential as an effective food preservative and paving the way for future research into peptide-based food preservation strategies.

## Graphical abstract

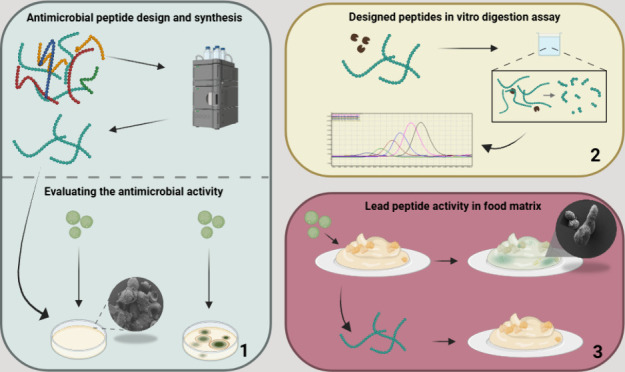

## 1 Introduction

The world faces many challenges demanding our attention to ensure a sustainable future of mankind. The main factors that are confronted are the impacts of greenhouse gas emission, depleting reservoirs of fossil fuels, and food loss and waste (FLW).^1^ According to the Food and Agriculture Organization (FAO) about 1.3 billion tons of food are lost at each stage of the food supply chain, and that amount has been increasing yearly.^2^ Food loss refers to food that is being lost during production, pre- and postharvest, and processing, whereas food waste refers to food discarded by food service providers, retailers, and consumers.^3^ Food waste occurs at the later stages of the food supply chain compared to food loss, resulting in more time, labor, and resources. As a result, the environmental impacts, especially the carbon footprint, was greater.^4^ Besides the ecological consequences, food waste has a major economic impact. In a 2020 report by the World Wildlife Fund (WWF), in Europe alone, in addition to being responsible for 15% of all greenhouse gas emissions, food waste is estimated at €143 billion per year.^5^ One of the major causes of food spoilage and FLW is as a result of fungal contamination; about 5–10% of food products and up to 30% of crops are being damaged by fungi^6,7^ .

Molds are multicellular filamentous fungi that form thin hair-like structures called hyphae.^8^ The major molds that take part in food spoilage belong to the genera and .^9^ Molds reproduce by forming conidia from their conidiophores, the spore-forming structure. These conidia are airborne and find new niches to germinate, thus spreading and enhancing the amount of spoiled food.^10^

To prevent mold contamination and food spoilage, food industries are using food preservatives, also known as antimicrobial agents, which extend the shelf life of food by preventing bacterial and fungal growth.^11^ The preservatives, mostly acid-based,^12^ can be harmful for human health.^13^ Furthermore, the pH is of utmost importance because some food preservatives (e.g., potassium sorbate) require only a narrow and acidic range of pH to be active;^14^ compelling the food industry to alter the food’s pH, thus affecting the food flavor and consumption.^15^

In recent years, there has been an increase in the demand by both consumers and manufacturers for natural and safe food preservatives.^12,16^ This has promoted the investigation of a variety of natural antimicrobial preservatives, such as nisin, a bacteriocin (antimicrobial peptide) produced by , which is effective against Gram-positive bacteria,^17^ active packaging, and use of herbs or oxygen absorbers.^16,18,19^ So far, these applications to extend shelf life and prevent fungal growth have been poorly applied.^20,21^

Antifungal peptides (AFPs) are a type of antimicrobial peptides (AMPs), which can inhibit the growth of fungi.^22^ AMPs are relatively short, positively charged, amphiphilic peptides.^23^ The origin of peptides may vary between plants, mammals, bacteria, and insects, and the number of peptides identified exceeds 5000 and continues to grow.^24^

In contrast to mammalian cells, fungi have a high content of phosphatidylinositol and phosphatidic acid, making their cell membrane negatively charged.^22,25^ Due to the AFPs positive charge, they interact with the negatively charged membrane, and due to the amphiphilic nature, the AFPs undergo membrane absorption, causing cell membrane damage.^26^ Moreover, interest in AMPs in a variety of fields is increasing due to their wide modes of action, which limits the risk of targeted microbes to develop resistance.^12^

The two model AMPs that were explored in this study are BP100 and P-113. BP100 (sequence: KKLFKKILKYL) is an AMP that was designed by combining two well-known AMPs: cecropin A and melittin,^27^ with strong activity against Gram-negative bacteria.^28^ P-113 (sequence: AKRHHGYKRKFH) is a histatin 5 derivative, a peptide secreted into the saliva,^29^ that had shown activity against both bacteria and yeasts.^30^

In this study, we hypothesized that several chemical modifications of the two model peptides, BP100 and P-113, will improve their activity and allow their application as food preservatives in a model food matrix, hummus (chickpea spread), which, due to its high-water activity, is susceptible to fungi spoilage.

## 2 Materials and Methods

### 2.1 Fungal and Bacterial Strains, Growth Media, and Growth Conditions

 A1163 was grown on potato dextrose agar (PDA) medium at 37 °C for 5 days, and then the conidia were scraped and suspended in PBS with 0.025% (v/v) Tween 20, counted using a hemocytometer, and kept at 4 °C. isolated from peanuts and Pe21 (both kindly provided by Dr. Edward Sionov, ARO, Israel) were grown on PDA medium at 25 °C for 5 days, and then the conidia were scraped and suspended in PBS with 0.025% Tween 20 and kept at 4 °C. RP and methicillin-resistant (MRSA) were grown on LB agar plates, and individual colonies were picked and grown in Mueller–Hinton (MH) broth overnight at 37 °C. was grown on PDA medium at 30 °C, and individual colonies were picked and grown in yeast extract peptone dextrose (YPD) media overnight at 30 °C. All fungal and bacteria strains were maintained at −80 °C in 50% (v/v) glycerol stock.

### 2.2 Solid-Phase Peptide Synthesis (SPPS)

Peptides were synthesized using Fmoc-based SPPS using a Liberty Blue peptide synthesizer. Synthesis was carried out on a Rink Amide resin (substitution of 0.6 mmol/g). The Fmoc protecting group was removed by 20% (v/v) piperidine in dimethylformamide (DMF). Each amino acid was coupled using hydroxychloride (Oxyma) and N,N′-diisopropylcarbodiimide (DIC). After the last amino acid coupling and deprotected, the resin was washed with DMF (3 × 5 mL), followed by washing with dichloromethane (DCM, 3 × 5 mL). Peptides were cleaved from the resin using cleavage cocktail [200 μL of deionized distilled water (DDW), 200 μL of tris­(2-carboxyethyl)­phosphine (TIPS), and 7.6 mL of trifluoroacetic acid (TFA)] and were shaken for 3 h. Later, cleaved peptides were filtered and precipitated from the TFA solution by the addition of cold ether and centrifuged for 10 min at 4 °C. Ether was then removed, and the pellet was dissolved in 20% (v/v) acetonitrile, frozen in liquid nitrogen, and lyophilized before reverse-phase high-performance liquid chronography (RP-HPLC) purification.

### 2.3 Peptide Purification

Crude peptides were purified by RP-HPLC (Shimadzu). The crude peptides were diluted to a final concentration of 40 mg/mL in dimethyl sulfoxide (DMSO). Semi-preparative Phenomenex Kinetex C18 (5 μm, 10 × 250 mm) was used with standard (RP)-HPLC conditions. MALDI-TOF spectrometry (Bruker Daltonik, Germany) was used to validate the synthesized peptide molecular weight. The purified peptides were frozen in liquid nitrogen, lyophilized, and stored at −20 °C.

### 2.4 Minimal Inhibitory Concentration (MIC) Assay

For filamentous fungi strains, serial dilutions of 100 μL ranged from 100 to 0.78 μM for each peptide in a 96 well plate using RPMI1640 with 165 μM MOPS. A total of 80 μL of inoculum was suspended with a conidia load of 5 × 10^3^ CFU/mL (final density of 2 × 10^3^ CFU/mL), and 20 μL of resazurin was diluted in RPMI1640 solution of 0.02% [final concentration of 0.0002% (w/v)]. In the control, 100 μL of treatment was replaced by the same volume with medium, while the negative control was 180 μL of the medium and 20 μL of resazurin solution. Plates were incubated at 37 °C for and 25 °C for and for 24 h. The results were quantified by measuring absorbance at 570 nm with a microplate reader (Tecan). The mean absorbance values were used in subsequent calculations. The growth of resazurin reduction and growth inhibition was calculated using the following normalization:

$$\mathrm{\% growth}=100-\mathrm{\% reduction}=\frac{\mathrm{abs of test agent}-\mathrm{abs of untreated control}}{\mathrm{abs of reduced resazurin}-\mathrm{abs of untreated control}}$$

The MIC was defined as the lowest concentration of the peptide that inhibited fungal growth by 90%, allowing for only 10% of the normal growth. For bacterial and yeast strains studies, serial dilutions of 100 μL ranged from 100 to 0.78 μM for each peptide in a 96 well plate using MH media for bacteria and YPD for yeast. A total of 100 μL of the microbe in suitable media (5 × 10^5^ CFU/mL) was inoculated in each well. In the untreated well (e.g., control), 100 μL of treatment was replaced by the same volume with medium, while in the negative control, media were only without microbes and peptides. After 24 h of incubation at 37 °C for bacteria and 30 °C for yeast, plates were read at 595 nm and MIC was defined as the lowest concentration of peptide that reduced the growth of the microbe to 10% growth.

### 2.5 Fungicidal Assay

A 10^4^ CFU/mL conidia load was incubated in yeast extract (YE) medium at 37 °C for and 25 °C for and at 200 rpm for 4 h, and 100 μL was transferred to 900 μL of PBS with peptide (final conidia load = 10^3^ CFU/mL). The negative control had no peptides, and for the positive control, we used H_2_O_2_ (final concentration of 8 μM). The treated conidia were incubated for an additional 4 h at the same temperature at 200 rpm. Once the second incubation was over, the inoculum suspension was diluted, and 100 μL of both the original and the dilution was spread on Petri plates with PDA with tetracycline (10 μg/mL) and chloramphenicol (25 μg/mL) medium to exclude the growth of bacteria. The plates were incubated at matching temperatures depending on the strain. After 48 h, the CFU/mL was calculated; CFU/mL = number of counted CFU × 10^–*n*^ (*n* = serial dilution).

### 2.6 Membrane Disruption Staining

A total of 10^7^ CFU/mL of was incubated in YE at 25 °C and 200 rpm for 4 h. A total of 100 μL was transferred to 900 μL of PBS with peptides (final concentration of conidia load = 10^6^ CFU/mL); control had no peptides. The treated conidia were incubated overnight under the same conditions. The following day, spores were introduced to 9 μL of propidium iodide (PI) to 100 μL of the overnight spore culture and tilted for 15 min. Images were obtained using an EVOS M5000 imaging system (Thermo Fisher Scientific).

### 2.7 Digestion Examination of the Peptides

Peptides were dissolved in 1 mL of 100 μM NH_4_HCO_3_ to a final concentration of 4 mM,^31^ followed by the addition of 1 μL chymotrypsin (1 mg/mL), and were incubated at 37 °C and 150 rpm. At each time point, 75 μL of the solution was transferred to 90 °C for inactivation of the enzyme. After the peptides were frozen in liquid nitrogen and lyophilized, they were dissolved in DMSO and were analyzed in HPLC using Analytic Phenomenex Luna C18 (5 μm, 4.6 × 250 mm) with standard RP-HPLC conditions. The area under curve (AUC) was calculated at each time point and normalized to time = 0.

### 2.8 Circular Dichroism (CD)

Peptides were dissolved in the indicated solution [DDW, 50% trifluoroethanol (TFE), and 90% TFE] to a concentration of 50 μM. A J-1100 spectropolarimeter (Jasco) was set with the following parameters: wavelength range of 190–260 nm, bandwidth of 1 nm, data pitch of 0.1 nm, and three accumulations. A baseline spectrum was recorded using a 0.1 cm quartz cuvette filled with buffer. The cuvette was then filled with the peptide solution, and the CD spectrum was recorded under the same conditions. The measurement was repeated 3 times. The baseline was subtracted from the spectrum of the peptides to obtain the corrected CD spectrum and analyzed to identify characteristic bands for α-helix, β-sheet, and random-coil structures.

### 2.9 Toxicity Assay on the Human Cell Line

HEK cells were grown at 37 °C with 5% CO_2_ in Deulbecco’s modifield Eagle’s medium (DMEM, Sigma) with 2 mM l-glutamine, 1% (v/v) penicillin and streptomycin (Penstrep), and 20% (v/v) fetal bovine serum. The day before the experiment, cells were detached by gentle scraping, counted with a hemocytometer to a concentration of 10^6^ cells/mL, and seeded into a 96-well microplate (Nunc, Thermo Scientific). The following day, serial dilutions from 100 to 0.78 μM for each peptide were introduced to the cells with DMEM lacking phenol red for 24 h. Later, 50 μL of 3-(4,5-dimethylthiazol-2-yl)-2,5-diphenyltetrazolium bromide (MTT, 0.5 mg/mL) was added to the wells for 2 h of incubation. At the end, 100 μL of DMSO was added for 30 min at 37 °C and 200 rpm, and the plate was read at 595 nm and normalized to the untreated cells.

### 2.10 Scanning Electron Microscopy (SEM)

A total of 2 × 10^7^ CFU/mL of conidia was incubated in YE medium at 25 °C and 200 rpm for 4 h; after incubation, 100 μL was transferred (final conidia load of 2 × 10^6^ CFU/mL) to 900 μL of PBS with 100 μM peptides for overnight incubation at 25 °C and 200 rpm. After incubation, samples were washed 3 times in PBS. The samples were suspended in 500 μL of 8% glutaraldehyde with 500 μL of PBS for 1 h, following 3 washes in PBS; simultaneously, slides were covered with 30 μL of polylysine at each side for 30 min. A total of 30 μL of each sample was added to each side of the slides and left at a moist box for 1 h. Samples were dehydrated using an increasing ethanol dilution series (25, 50, 70, 90, 95, and 100%) each for 5 min twice. Afterward, samples were dried to the critical point dehydration (CPD) with liquid CO_2_ using a Quorum K850 critical point dry system. Lastly, samples were coated with a 2 nm layer of Au/Pd particles using a Quorum Q150T ES turbomolecular pumped sputter, before using JEOL JSM-7800f-HR-SEM.

### 2.11 Food Matrix Examination

Cooked and frozen chickpeas were purchased from a local store and were kept at −4 °C. For each experiment, 100 g was taken out and boiled in autoclaved DDW while covered for 2 h on a hot plate. Chickpeas were than blended (blender was sterilized using 70% ethanol and UV) with 50 mL of sterile DDW and divided into 1 mL portions; 200 CFU/mL of and 25 μL of 8 mM peptides (final concentration of 200 μM) were added, with the control having the same volume of autoclaved DDW, with incubation at 25 °C. At each time point, samples were diluted and both the original and dilutions were spread on PDA Petri plates in triplicates with tetracycline (10 μg/mL) and chloramphenicol (25 μg/mL) medium. The plates were also incubated at 25 °C, and colonies were counted after 48 h to calculate the growth of .

### 2.12 Statistical Analysis

Statistical analysis was performed using one-way ANOVA. When significant differences were found, Dunnett’s post hoc test was applied to compare treatment groups to the control. Differences were considered significant at a *p* value of <0.05.

## 3 Results

### 3.1 Peptide Design and Synthesis

BP100 and P-113 are AMPs that have been previously shown a broad activity against a variety of microorganisms, such as yeast and bacteria;^30,32,33^ despite that, their activity against molds have been poorly studied so far. Here, we explored their activity and performed several chemical modifications to improve their efficacy against filamentous fungal growth and survival. BP100 is predicted to form an amphiphilic helix^34^ upon binding to membranes; the helical wheel projection is shown in Figure 1B. Here, we substituted N-terminal lysine with different hydrophobic amino acids in order to elongate the hydrophobic face of the α helix in attempts to improve the efficacy^35,36^ (Figure 1). Moreover, Rothstein et al.^30^ showed that substituting the histidine amino acids in P-113 (AKRHHGYKRKFH) (Figure S1) did not substantially affect the cell killing activity against .^30^ Hence, we replaced the histidine amino acids with lysine to explore the effect of the free amino group of lysine on the activity (Figure S1).

**Figure 1:**
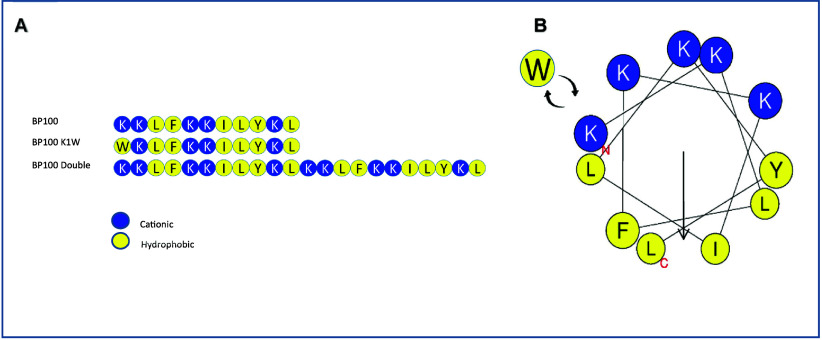
(A) Peptides sequences. (B) Helical wheel projection of BP100. The hydrophobic amino acids are shown in yellow, and the positive charge amino acids are in blue. The plot was obtained using the HeliQuest server.^36^

Previous studies have shown improved activity with increased peptide chain length.^30,37^ Therefore, in addition to altering the amino acids, we studied the effects of the chain length increase in both peptides. This was done by synthesizing the original peptide sequence 2 or 3 times (Figure S1); an example for BP100 and its doubled form (i.e., BP100 Double) can be seen in Figure 2A.

**Figure 2:**
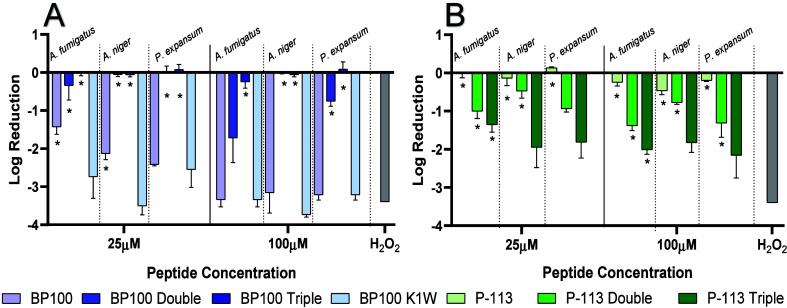
Fungicidal activity of BP100 and P-113 and their derivatives. Spores were counted using a hemocytometer and diluted to 2 × 10^3^ CFU/mL. After 4 h of incubation with the selected peptides in two different concentrations (25 and 100 μM), samples were diluted and both original and diluted samples were plated. Colonies were counted after 48 h of incubation and are presented as the log reduction of CFU/mL. For the positive control, fungi were treated with 8 μM H_2_O_2_ and were completely eradicated. The experiments were repeated 3 times (biological repeats) in triplicate (average ± SEM). Statistical analysis was conducted using Dunnett’s multiple comparison test. Asterisks (∗) indicate statistically significant results with a *p* value of less than 0.05 (α < 0.05).

### 3.2 Antimicrobial Activity

We have performed a comprehensive and comparative antimicrobial study. We have examined the growth inhibitory activity of the peptides toward model filamentous fungi and bacteria. Table 1 summarizes the ability of the AMPs to prevent the growth of both filamentous fungi and bacteria using a MIC study. When P-113 was synthesized as three copies in tandem (i.e., P-113 Triple) (Figure S1), it shows higher antimicrobial activity compared to the single-sequence P-113 original peptide. In contrast to P-113 elongation, increasing the length of BP100 resulted in a decrease activity against bacteria while simultaneously diminishing its effectiveness against fungi. Interestingly, changing the first amino acid in BP100 peptide sequence from lysine to tryptophan (i.e., BP100 K1W) showed stronger antimicrobial activity toward all tested microbes, expect , in comparison to BP100 (Table 1).

**Table 1:**
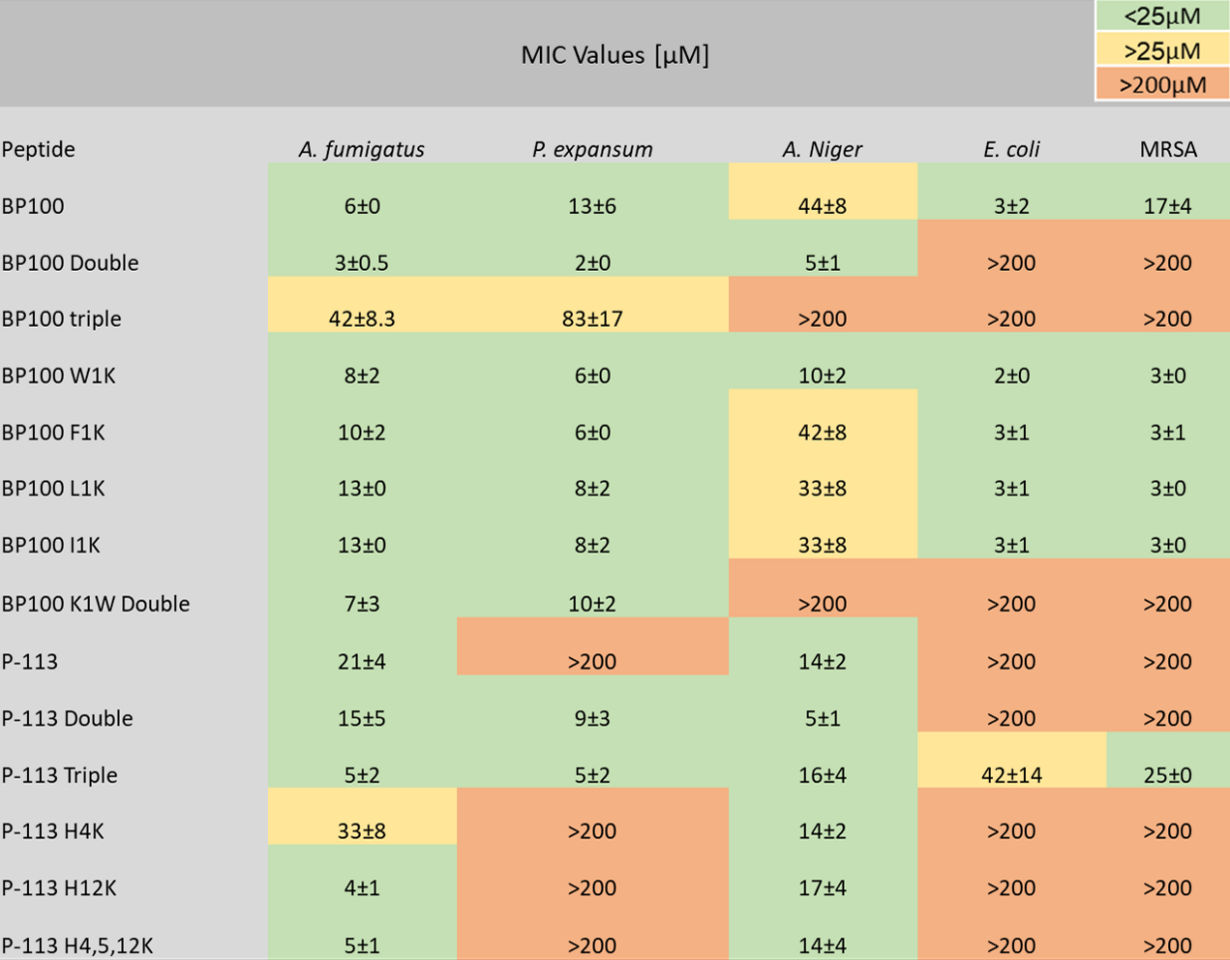
MIC Values^a^

a Bacteriostatic and fungi-static activity of BP100 and P-113 and their modifications. The experiments were repeated 3 times (biological repeats) in triplicates (average ± SE).

Moreover, P-113 Triple, BP100, and BP100 K1W showed inhibitory activity against the yeast (Figure S5).

In the next step, we evaluated selected peptides for their fungicidal activity (Figure 2). Fungicidal activity of P-113 and its derivatives (Figure 1A) supports the observed MIC values (Table 1). When we increased the P-113 chain length, the fungicidal and fungal growth inhibition activity on all tested isolates has increased. In addition, there was high fungicidal activity for both BP100 and BP100 K1W at 100 μM; strong fungicidal activity occurs for BP100 K1W at 25 μM as well. BP100 Double and BP100 Triple both had low to no fungicidal activity (Figure 2B).

### 3.3 Antimicrobial Peptide Mode of Action

Membrane damage is a common mechanism of action of many AMPs when interacting with the microbial cells; this damage often leads to the disruption of cellular integrity, resulting in cell death.^38^ To further study the mechanism of action of our model AMPs and their derivatives, we exposed spores to different peptides, followed by PI staining to visualize the membrane damage using a fluorescence microscope (EVOS). Our results confirmed the membrane damage caused by these peptides (Figure S2).

Furthermore, a qualitative evaluation of membrane damage was conducted using SEM. Figure 3 shows the damage of spores after exposure to the following peptides: BP100, BP100 K1W, P-113, and P-113 Triple (100 μM). It can be observed that BP100 K1W had the most significant effect on the spore’s morphology. On the contrary, P-113 did not show any effect on the spores compared to the untreated (i.e., control) spores, as expected from the MIC and fungicidal assay results (Table[Table tbl1] and Figure 2B).

**Figure 3:**
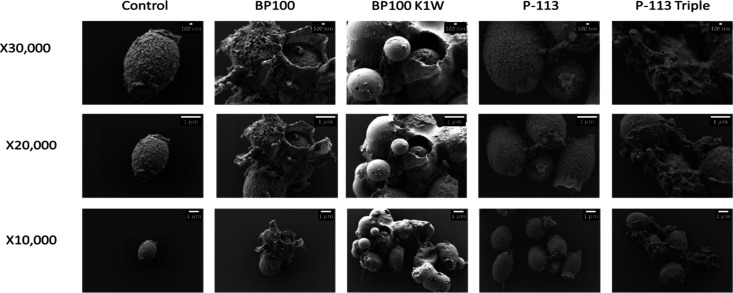
SEM images of . cells were treated for 24 h at 25 °C and 200 rpm with the indicated peptide (100 μM control, BP100, BP100 K1W, P-113, and P-113 Triple). Imaging was preformed using JEOL JSM-7800f-HR-SEM.

To design a novel food preservative, it is important to develop selective agents that will not affect the host. We evaluated the peptide cytotoxicity against HEK mammalian cells using the MTT assay (Figure S3). All tested peptides exhibited minimal to no cytotoxic effects, even at the highest concentrations examined. This finding emphasizes the high selectivity of the peptides toward bacteria and fungi over mammalian cells. This is a critical requirement for safe applications in food systems and human health. Given the importance of food preservation, these results provide an encouraging basis for further development.

### 3.4 Enzymatic Digestion

To understand the functional properties and stability of the peptides, we characterized their secondary structure. Peptides were examined in the CD instrument under three different conditions: (1) DDW, (2) 50% TFE, and (3) 90% TFE (Figure S4). Figure 4A presents the CD spectra of the tested peptides in DDW, indicating that all peptides expect BP100 Double and BP100 Triple are unstructured (random coil). Peptides BP100 Double and BP100 Triple are the only peptides folded into an α-helix conformation in DDW. As the peptides are intended to serve as food preservatives and undergo consumption, we examined their potential to be enzymatically degraded. Figure 4B shows that chymotrypsin digested all of the peptides very fast, except BP100 Double and BP100 Triple that were less prone for digestions.

**Figure 4:**
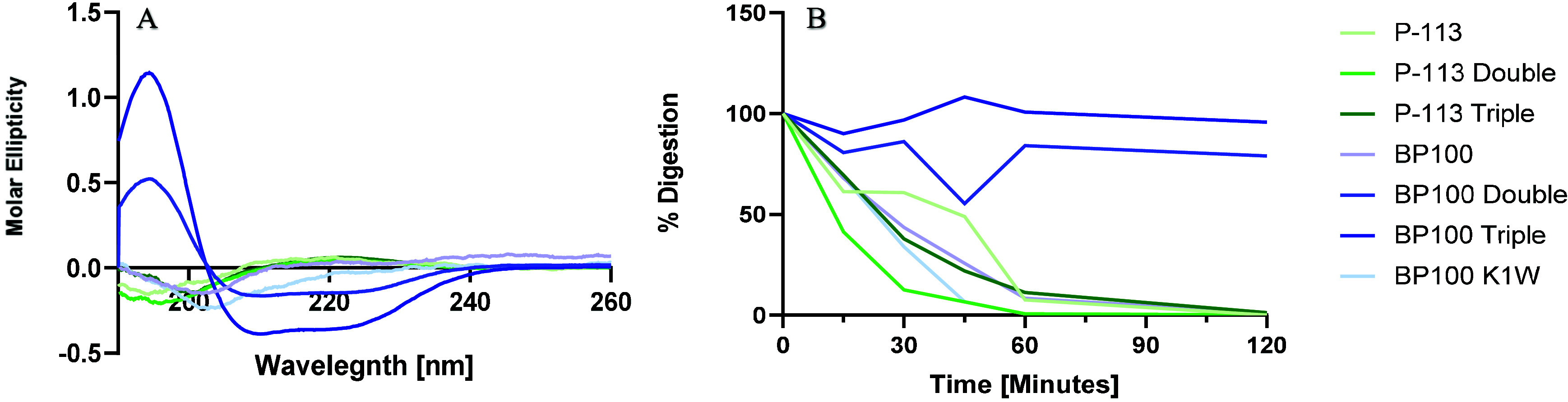
Peptide’s secondary structure and digestibility. (A) CD spectra of the peptides (50 μM) in DDW was measured using a J-1100 spectropolarimeter (Jasco), with the following parameters: wavelength range of 190–260 nm, bandwidth of 1 nm, data pitch of 0.1 nm, and three accumulations. A baseline spectrum was recorded using a 0.1 cm quartz cuvette filled with DDW and subtracted from the peptide spectrum to obtain the corrected CD spectrum. (B) Breakdown of the peptides: P-113, P-113 Double, P-113 Triple, BP100, BP100 Double, BP100 Triple, and BP100 K1W by chymotrypsin, quantified by analytical HPLC. The area under the curve (AUC) was calculated at each time point and normalized to *t* = 0. The *y* axis ranges from 100%, meaning not digested at all, to 0%, meaning fully digested.

### 3.5 Food Matrix

Following the evaluation of the enzymatic digestion, selectivity, and antimicrobial activity, we tested the lead peptides: P-113, P-113 Triple, BP100, and BP100 K1W activity in hummus (chickpea spread) as a model for the complexed food matrix. There is an increase in the popularity of the hummus spread in both United States and Europe,^39^ and due to its high-water activity, it is susceptible to contamination.^40^ As summarized in Figure 5, P-113 and its modification did not prevent the growth of in the spread; fungi treated with P-113 and P-113 Triple grow at the same rate as the untreated fungi, ending up with 10^8^ CFU/mL. While BP100 had an impact on growth, growing slower than the untreated fungi and reaching 10^6^ CFU/mL, BP100 K1W showed strong inhibitory activity against the proliferation of the fungi in hummus for 7 days with little to no growth. Thus, this demonstrates the great potential of BP100 K1W for further studies as food preservatives while also highlighting the crucial step of chemical modification of AMPs to improve their activity.

**Figure 5:**
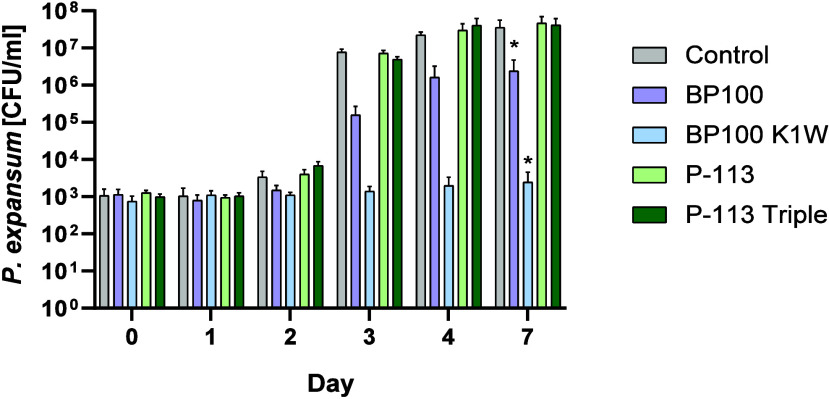
Log reduction of growth with lead peptides in the food matrix. Hummus was inoculated with (200 CFU/mL) and then was treated with the indicated peptides at 200 μM. Every day a sample (100 μL) was taken and diluted, and both the original and dilutions were plated on Petri plates with tetracycline (10 μg/mL) and chloramphenicol (25 μg/mL) medium to follow the proliferation of . The experiments were repeated 3 times (biological repeats) in triplicates (average ± SEM). Statistical analysis was conducted using Dunnett’s multiple comparison test. Asterisks (∗) indicate statistically significant results with a *p* value of less than 0.05 (α < 0.05).

## 4 Discussion

Our aim in this study was to investigate the potential of BP100 and P-113 and their modified derivatives to inhibit the growth of food spoilage fungi and, by that, to serve as food preservative agents in hummus as a food matrix model. Initially, we evaluated the antifungal activity of these peptides. When testing various chain lengths of P-113 across a variety of microbial strains, the findings were consistent with those reported by Lin et al.^37^ Moreover, Zamora-Carreras et al.^35^ demonstrated that substituting lysine with alanine in BP100 did not alter the antimicrobial activity of the peptide. To further explore ways to improve the peptide activity, we substituted N-terminal lysine with the hydrophobic amino acids: (1) phenylalanine, (2) tryptophan, (3) leucine, and (4) isoleucine, aiming to increase membrane damage and antimicrobial activity. As demonstrated, the increase of hydrophobicity had a positive effect on the inhibitory activity, with tryptophan substitution having the best activity (Table 1); these findings are consistent with previous studies.^41,42^ In addition, from the activity presented in Table 1, BP100, BP100 K1W, and P-113 Triple showed fungistatic activity against (Figure S5), demonstrating their broad activity against yeast, molds, and bacteria, a crucial test for food preservative.

SEM images demonstrated the enhanced effect of P-113 Triple, whereas the original P-113 did not show any visual damage to the fungal membrane (Figure 3). Moreover, a strong BP100 K1W effect was observed compared to the original BP100 peptide when N-terminal lysine was replaced by tryptophan (Figure 2B). These images suggest that those peptides’ primary mechanism of action may involve membrane pore formation and structural disruption, a well-known mechanism of AMPs.^43–45^ Moreover, we hypothesize that the increased hydrophobic character of the BP100 K1W variant led to enhanced self-assembly of the peptides, thereby improving its activity.^46^ Additionally, we suggested that the extended length of the P-113 Triple variant enabled self-interactions that enhanced its activity, although further mechanistic studies are needed to confirm these hypotheses. As was reportedin previous studies, amphipathic α-helical structuresd peptides, are highly selective.^45^ Here, in addition to their high antimicrobial activity, BP100 K1W and P-113 Triple demonstrated low to no cytotoxicity toward the mammalian cell line (Figure S3). This not only confirms their safety but also underscores their selectivity. Thus, they are presented as prime candidates for further studies not only to serve as food preservatives but also in pharmaceutical studies.

Following a new food preservative, we would expect that these agents will be fully digested in the gastric system without affecting the consumers’ microbiome. To investigate the peptide enzymatic degradation, we have conducted an *in vitro* assay using chymotrypsin. This enzyme was chosen due to its activity on aromatic amino acids.^47^ Moreover, we assume trypsin will have similar activity because it cleaves lysine amino acid,^48^ which is abundant in AMPs. After testing the peptide digestibility by chymotrypsin (Figure 4B) and examining their secondary structure (Figure 4A), we found that only BP100 Double and BP100 Triple peptides were not digested and preserved an α-helix structure (Figure 4). From this, we hypothesize that the ability of the longer peptides to fold into a stable α helix might protect them from digestion, which stands with the results seen by past studies, indicating that the secondary structure can prevent enzymatic cleavage.^49,50^ Most importantly, we observed that the lead peptides, BP100 K1W and P-113 Triple, were fully degraded within a short time frame, indicating their potential to be easily digested and utilized as food preservatives.

Lastly, BP100 K1W, P-113 Triple, and their derivatives were added to hummus that was contaminated with , as there are past reports on finding growth in the spread.^51^ While P-113 and P-113 Triple did not show any inhibitory activity toward the fungi, BP100 K1W demonstrated strong inhibition of the spoilage in the hummus up to 7 days, demonstrating its ability to extend the shelf life of the hummus (Figure 5), thus highlighting its potential as an effective, selective, and safe food preservative. However, despite the promising results, it is important to note that future studies are needed to assess the peptides’ resistance to heat and their stability under various conditions. Moreover, upscaling of peptide synthesis still remains a significant challenge.^52^ Nonetheless, our findings demonstrate that BP100 K1W and P-113 Triple peptides possess strong antifungal activity, broad selectivity, and low cytotoxicity and are fully degraded in the digestive system model. Making them promising candidates for use as food preservatives. Thus, this paved the way for future research into peptide-based food preservation strategies.

## Supplementary Material



## Notes

BP100 and P-113 modifications (Figure S1), pictures of membrane damage in spores following peptide treatment using PI staining (Figure S2), cytotoxicity evaluation of peptides on HEK cells via the MTT assay (Figure S3), secondary structure characterization of the peptides under different TFE concentrations (Figure S4), and survival assay of under peptide exposure (Figure S5) (PDF)
