# Supplementary material for: Modifying Antifungal Peptides as Safe Food Preservatives
Source: J Agric Food Chem. 2025 Jul 10;73(29):18420–7. doi: 10.1021/acs.jafc.5c02065 (PMC12291464; doi:10.1021/acs.jafc.5c02065)
Supplement: Supplementary file 1 [file jf5c02065_si_001.pdf]

Modifying Anti-fungal Peptides as Safe Food Preservatives

Supporting information

Eyal Simons[1], Idan Yakir[1,2], Einav Malach[1], Aygun Israyilova[3], Zvi Hayouka[1,2]*

[1] Institute of Biochemistry, Food Science and Nutrition, The Robert H. Smith Faculty of Agricultural, Food & Environment, The Hebrew University of Jerusalem, Rehovot, 76100, Israel. [2] Singapore-HUJI Alliance for research and enterprise (SHARE), The Cellular Agriculture (CellAg) Programme, Campus for Research Excellence and Technological Enterprise (CREATE), 138602 Singapore. 3. Baku State University, Biomedical materials, Baku, Azerbaijan.

Corresponding author email: zvi.hayouka@mail.huji.ac.il

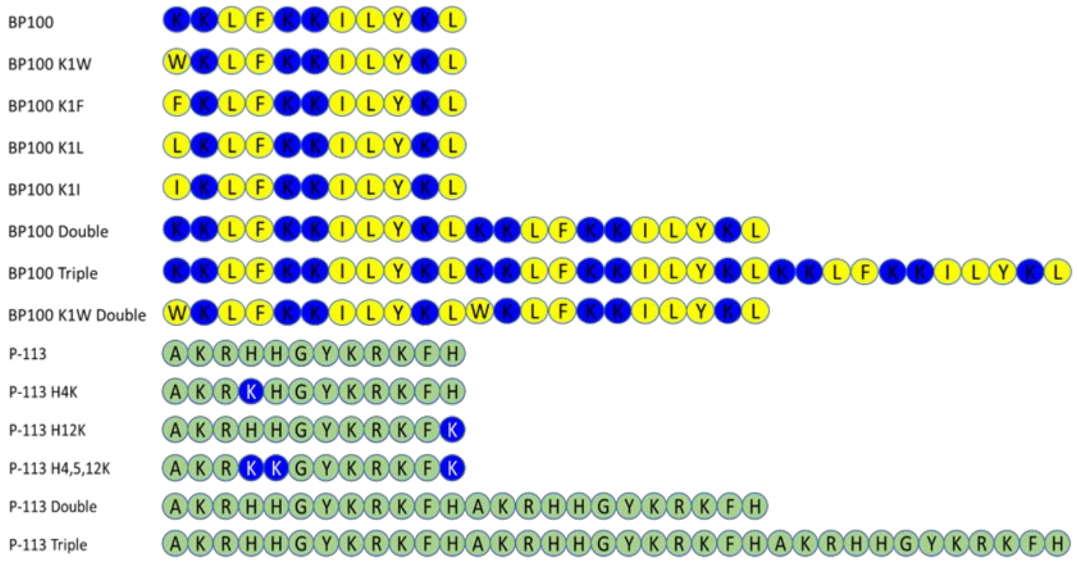

S1. Modifications of BP100 & P-113. Peptides that were designed, synthesized and purified for the current study.

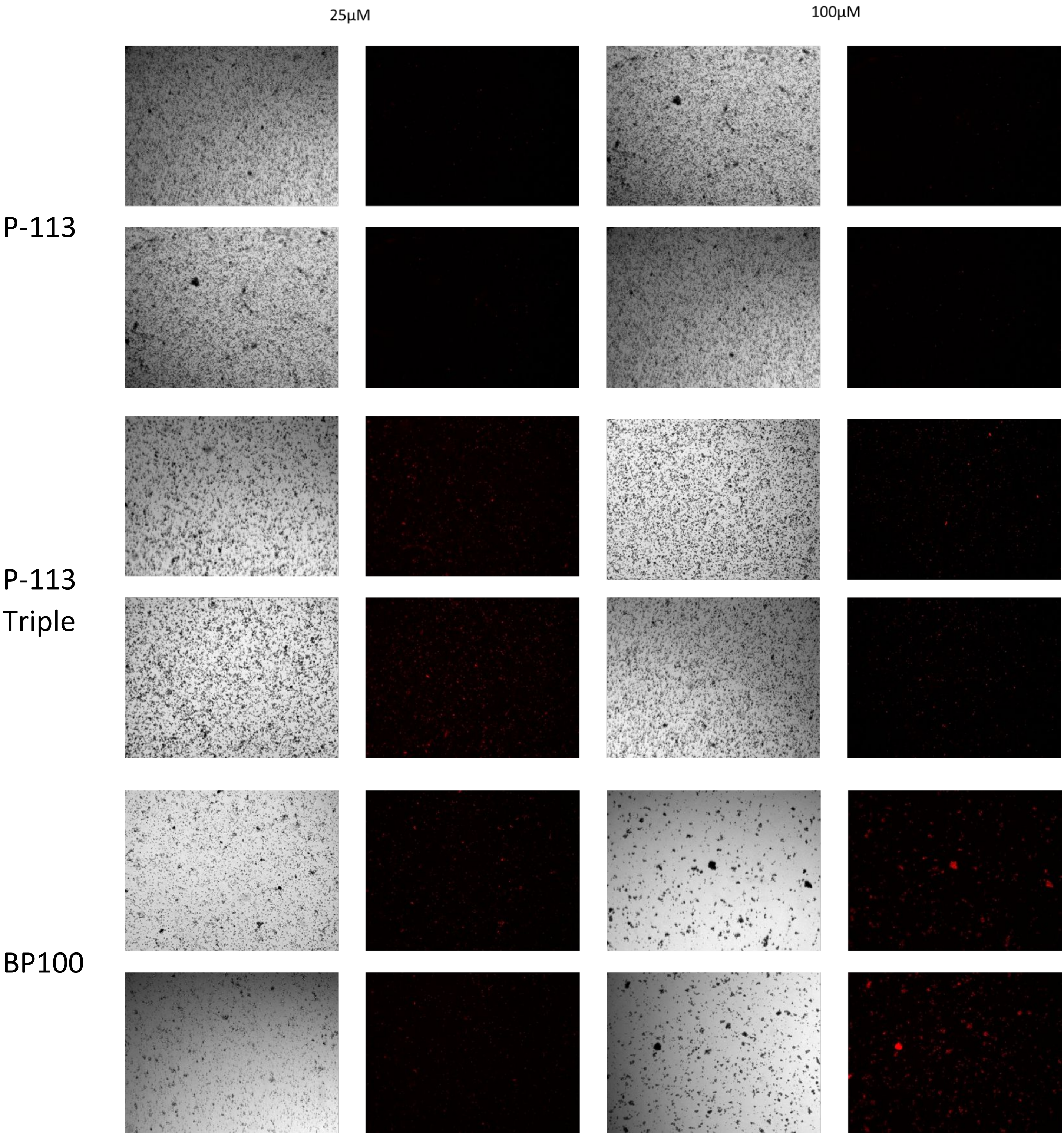
25µM
100µM
P-113
P-113
Triple
BP100

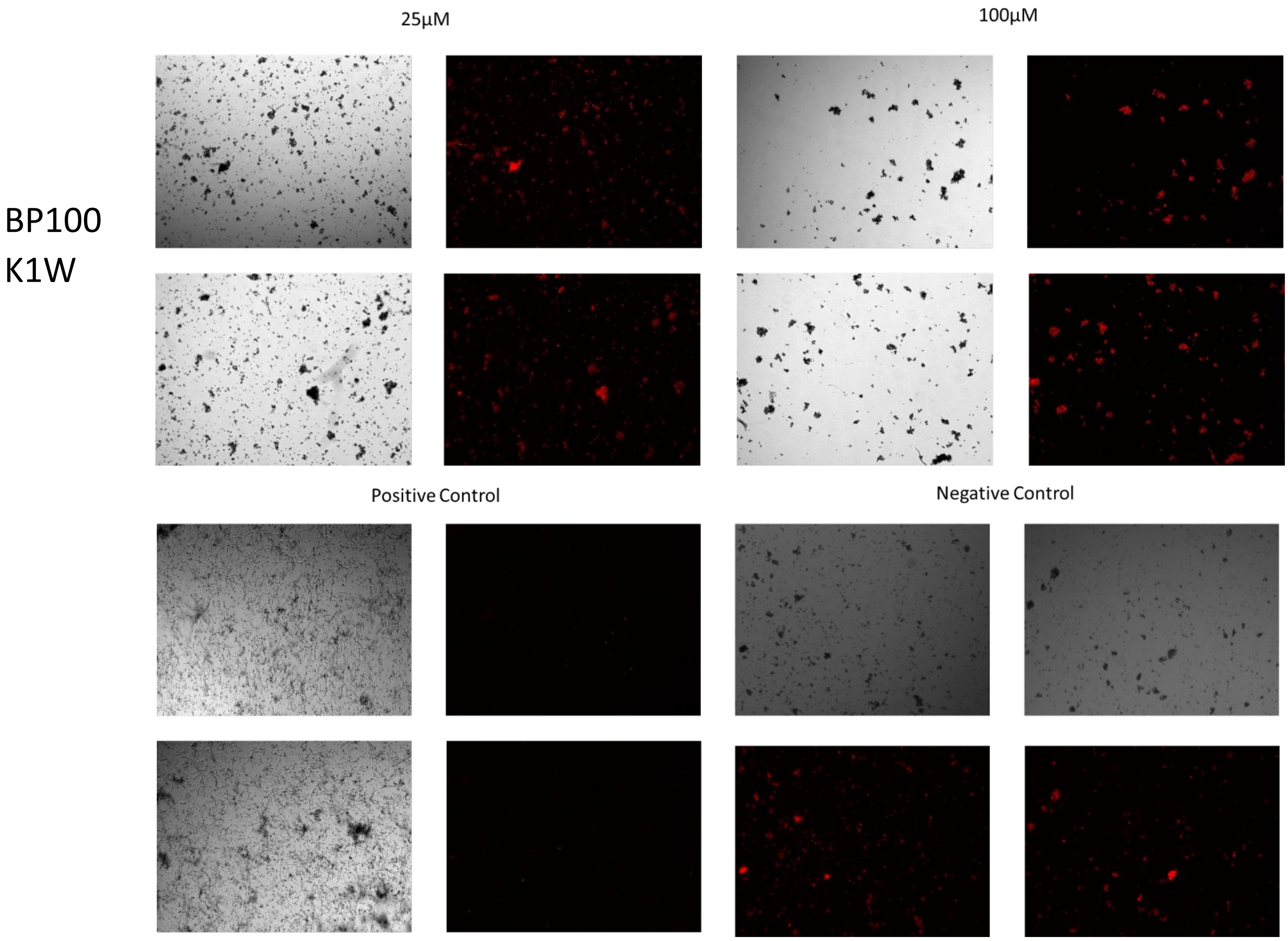

S2. Assessment of Membrane Damage in *P. expansum* spore upon peptides treatment. Spores were counted using hemocytometer and diluted to 10^6 CFU/mL. Spores were treated for 24 hours at 25°C, 200rpm with 25µM & 100µM of the indicated peptides: BP100, BP100 K1W, P-113 and P-113 Triple; Negative control was 8µM H_2O_2 and positive control was equal volume of DDW. Following incubation, spores were introduced to 9µl of propidium iodide (PI) to 100µl of the overnight spore culture. Images were obtained using EVOS M5000 imaging system (Thermo Fisher Scientific).

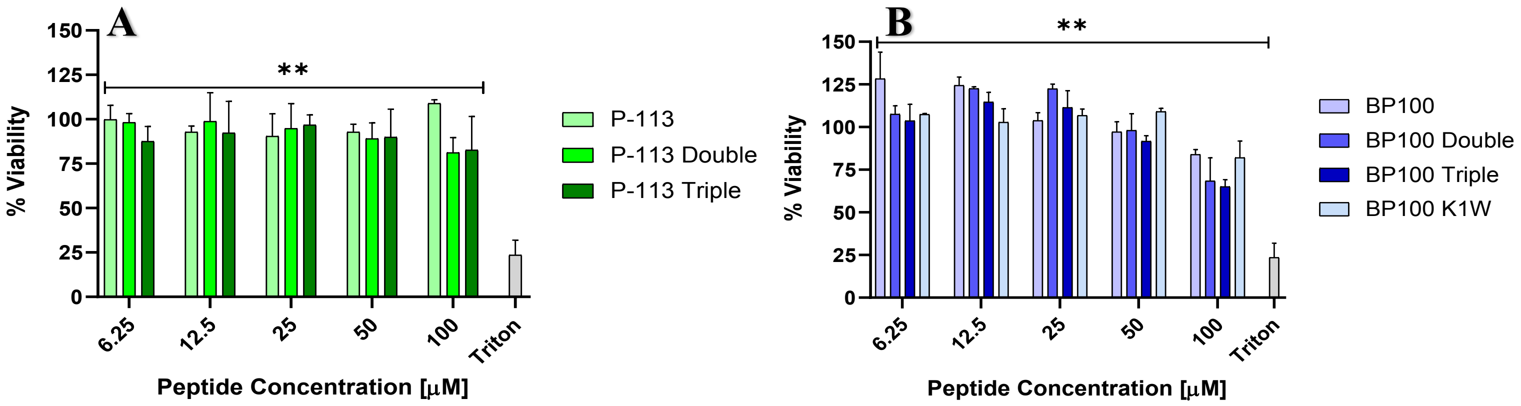

Supplementary 3. Cytotoxicity examination. HEK cells were cultured in DMEM with L-glutamine, PenStrep, and fetal bovine serum at 37°C and 5% CO_2. The day before the experiment, cells were detached, counted (10^6 cells/ml), and seeded into a 96-well plate. Peptides at concentrations from 100µM to 0.78µM were added for 24 hours; 1% Triton was used for control. Cell viability was assessed using the MTT assay; after adding 50µl of MTT and incubating for 2 hours, 100µl DMSO was added for 30minutes at 37°C, 200rpm. Next, absorbance was measured at 595nm and normalized to untreated controls. The experiments were repeated three times (biological repeats) in triplicates (average ± SEM). Statistical analysis was conducted using the Dunnett's multiple comparison test against the control. Asterisks (**) indicate statistically significant results with a *p*-value of less than 0.001 ($\alpha < 0.001$).

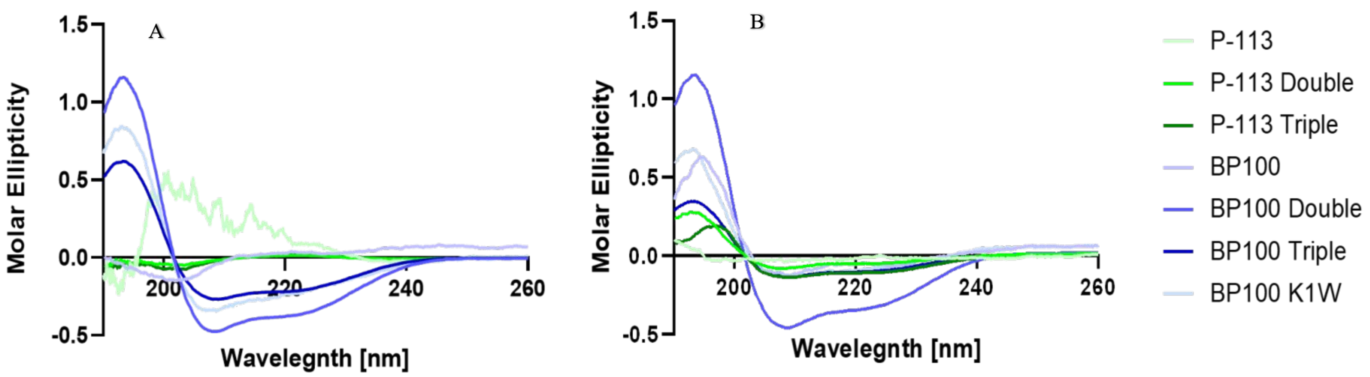

S4. Characterizing the secondary structure of the peptide in the presence of Trifluoro ethanol (TFE). CD spectra of the peptides: P-113, P-113 Double, P-113 Triple, BP100, BP100 Double, BP100 Triple and BP100 K1W (50µl) in (A) 50% TFE and (B) 90% TFE was recorded usingJ-1100 spectropolarimeter (Jasco) with the following parameters: wavelength range 190-260nm, bandwidth 1nm, data pitch 0.1nm and three accumulations. A baseline spectrum was recorded using a 0.1cm quartz cuvette filled with buffer (50% TFE & 90% TFE respectively) and subtracted from the peptides spectrum to obtain the corrected CD spectrum.

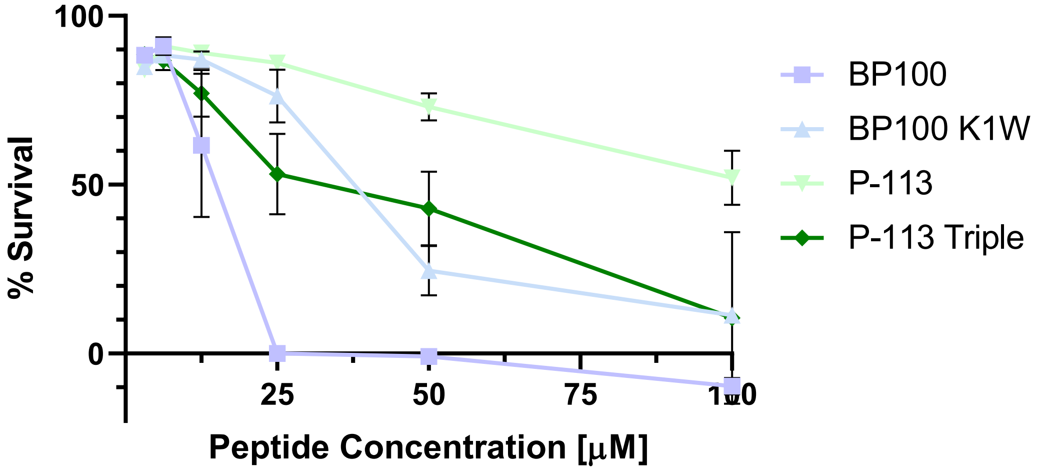

S5. Survival curve for *S. cerevisiae*. Serial dilutions of each peptide: BP100, BP100 K1W, P-113 and P-113 Triple were made in 96 well plate; followed by adding *S. cerevisiae* ($5X10^5$CFU/mL) for 24h at 30^oC. Plates were than read at 595nm O.D and normalized to untreated yeast. The experiments were repeated three times (biological repeats) in triplicates (average ± SEM)
